# An Improved Math Word Problem (MWP) Model Using Unified Pretrained Language Model (UniLM) for Pretraining

**DOI:** 10.1155/2022/7468286

**Published:** 2022-07-14

**Authors:** Dongqiu Zhang, Wenkui Li

**Affiliations:** ^1^Education Science Department of Nanjing Normal University, Nanjing 210000, China; ^2^Information Engineering Department of Suihua University, Suihua 152000, China

## Abstract

Natural Language Understanding (NLU) and Natural Language Generation (NLG) are the general methods that support machine understanding of text content. They play a very important role in the text information processing system including recommendation and question and answer systems. There are many researches in the field of NLU such as Bag of words, N-Gram, and neural network language model. These models have achieved a good performance in NLU and NLG tasks. However, since they require lots of training data, it is difficult to obtain rich data in practical applications. Thus, pretraining becomes important. This paper proposes a semisupervised way to deal with math word problem (MWP) tasks using unsupervised pretraining and supervised tuning methods, which are based on the Unified pretrained Language Model (UniLM). The proposed model requires fewer training data than traditional models since it uses model parameters of tasks that have been learned before to initialize the model parameters of new tasks. In this way, old knowledge helps new models successfully perform new tasks from old experiences instead of from scratch. Moreover, in order to help the decoder make accurate predictions, we combine the advantages of AR and AE language models to support one-way, sequence-to-sequence, and two-way predictions. Experiments, carried out on MWP tasks with 20,000+ mathematical questions, show that the improved model outperforms the traditional models with a maximum accuracy of 79.57%. The impact of different experiment parameters is also studied in the paper and we found that a wrong arithmetic order leads to incorrect solution expression generation.

## 1. Introduction

The basic research of natural language processing (NLP) is human-computer language interaction, which reflects human language with algorithms that can be understood by machines. NLP can perform a vast array of tasks such as text summarization, generating completely new pieces of text, and predicting what word comes next, among others. The core is a language model (LM) based on statistics. Honestly, these LMs are a crucial first step for most of the advanced NLP tasks. This paper will begin from basic LMs that can be created with a few lines of Python code and move to state-of-the-art language models that are trained using humongous data and are being currently used by the likes of Google, Amazon, and Facebook, among others. LMs are the probability distribution of a sequence of words, which can quantitatively evaluate the possibility of a string of characters. LMs are used in speech recognition, machine translation, part-of-speech tagging, parsing, optical character recognition, handwriting recognition, information retrieval, and many other daily tasks. Its ability to model the rules of a language as a probability gives great power for NLP-related tasks. The general process includes a process of predicting the back words. And then, the probabilities of all words are used to evaluate the possibility of the existence of the text. There are two types of LM: Statistical Language Models and Neural Language Models [1–4]. Statistical LMs use traditional statistical techniques like *N*-grams, Hidden Markov Models (HMM), and certain linguistic rules to learn the probability distribution of words. For example, Mezzoudj and Benyettou [5] augment naive Bayes models with statistical n-gram language models to address the shortcomings of the standard naive Bayes text classifier. In the work of [6], they propose a fast and simple algorithm for training NPLMs based on noise-contractive estimation, a newly introduced procedure for estimating un-normalized continuous distributions. Experiment results show that the model reduces the training times by more than an order of magnitude without affecting the quality of the resulting models. The algorithm is also more efficient and much more stable than importance sampling because it requires far fewer noise samples to perform well.

However, the estimation will be difficult in practice if the text is very long. Thus, there is a simplified method: the *N*-grams model. In the *N*-grams model, the conditional probability of the word is estimated by calculating the first *N* words of the current word. Unigram, bigram, and trigram are the commonly used *N*-grams models. Typed character *N*-grams reflect information about their content and context. According to previous research, typed character *N*-grams improve the accuracy of authorship attribution [7, 8]. However, the problem of data sparseness and inaccuracy gets worse with the larger text in these models. In order to solve the problem of data sparseness when estimating probability with the *N*-grams model, researchers try to use neural networks to study the language model, such as UniLM and TransFormer.

This paper proposes a semisupervised approach based on UniLM, which uses unsupervised preview and supervised tuning for language processing tasks. The goal of this approach is to learn a universal representation that requires very little adaptive adjustments when migrating to various downstream tasks. The training process of the algorithm is divided into two stages: the first stage uses language modeling targets on unlabeled data to learn the initial parameters of the neural network; the second stage uses the corresponding supervised targets to adapt these parameters to the target task. Moreover, to evaluate the performance of our model in comparison with other models, we carried out a highly challenging deep QA task on a large-scale and template-rich dataset of Math Word Problems Math23K [9]. The results show it has a maximum accuracy of 79.57%.

There are three advantages and contributions of the proposed model: (1) Although there are three language model tasks in the pretraining process, we do not need to train the three models separately because the parameters of the transformer are shared. Thanks to the self-attention masking of UniLM. (2) Parameter sharing makes the learned text representation more universal because these parameters are jointly optimized with different language models. It also alleviates the problem of over-fitting on a specific language model task. (3) The proposed model is suitable for both NLU and NLG problems.

## 2. Related Work

In 2000, researchers first put forward the idea of neural networks to study language models [10–12]. Until 2011, Collobert and Weston [13] used a simple deep learning model to achieve SOTA results in NLP tasks such as named entity recognition NER, semantic role tagging SRL, and part-of-speech tagging POS-tagging. More and more researchers focus on the methods based on deep learning. In 2013, the word vector represented by Word2vec [14] and Pennington et al. [15] became popular. More research has explored to improve the ability of language models from the perspective of word vectors, and focused on the semantics of words and context. In 2014, Kim proposed a TextCNN [16] model based on pretrained Word2vec for sentence classification tasks. In 2016, Joulin et al. [17] proposed a simple and lightweight deep learning model for text classification: FastText. The architecture is similar to the Word2vec CBOW model proposed by Rong et al. [18]. Experiment results show that FastText can achieve a good performance with efficiency.

**Figure 1:**
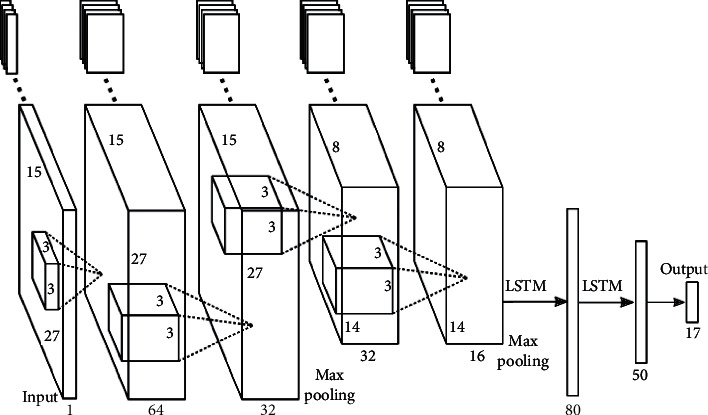
Illustration of the CNN-RNN-based defense architecture.

In addition, researchers have tried to use various mechanisms to optimize the ability of language models such as CNN, RNN, and Transormer [19, 20]. The CNN-LSTM architecture involves using Convolutional Neural Network (CNN) layers for feature extraction on input data combined with LSTMs to support sequence prediction. As shown in Figure 1, a common CNN-LSTM model is composed of a cell, an input gate, an output gate, and a forget gate. The cell remembers values over arbitrary time intervals and the three gates regulate the flow of information into and out of the cell. CNN-LSTM networks are well-suited to classifying, processing, and making predictions based on time series data, since there can be lags of unknown duration between important events in a time series. In a CNN, a convolution operation is used to obtain multiple feature maps. Then, it extracts key information for classification by filtering noise information through the pooling operation. Among them, pretraining combined with downstream task fine-tuning methods is the most eye-catching trend. In [21], for example, they investigate the benefits of integrating CNNs and LSTMs and report obtaining improved accuracy for Arabic sentiment analysis on different datasets. Additionally, we seek to consider the morphological diversity of particular Arabic words using different sentiment classification levels.

In AI, pretraining imitates the way human beings process new knowledge using model parameters of tasks that have been learned before to initialize the model parameters of new tasks. In this way, old knowledge helps new models successfully perform new tasks from old experience instead of from scratch. In recent years, EMLo, GPT, and BERT frequently refreshed the SOTA result [22]. For example, [23] trained a BERT language understanding model for the Italian language (AlBERTo). In particular, AlBERTo is focused on the language used in social networks, specifically on Twitter. To demonstrate its robustness, we evaluated AlBERTo on the EVALITA 2016 task SENTIPOLC (SENTIment POLarity Classification) obtaining state-of-the-art results in subjectivity, polarity, and irony detection on Italian tweets.

**Figure 2:**
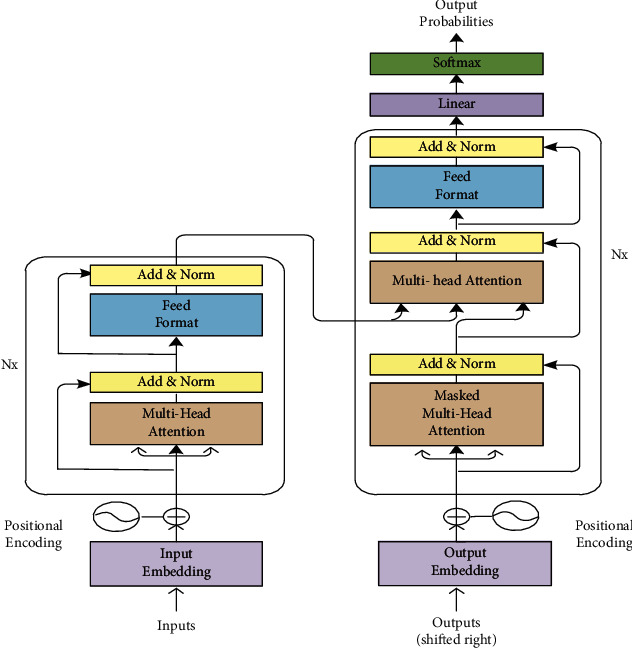
The transformer model architecture.

Transformer [24], which is based on the attention mechanism, completely abandoned CNN and RNN, and only captured the global relationship between the input and the output. As shown in Figure 2, the transformer architecture is composed of two parts: Encoder and Decoder. The encoder is on the left and the decoder is on the right. Both the encoder and decoder are composed of modules that can be stacked on top of each other multiple times, which is described by *N*_*x*_ in the figure. We see that the modules consist mainly of multi-head attention and feed forward layers. The inputs and outputs (target sentences) are first embedded into an n-dimensional space since we cannot use strings directly.

Transformer architectures have facilitated building higher capacity models and pretraining has made it possible to effectively utilize this capacity for a wide variety of tasks. The effectiveness of transfer learning has given rise to a diversity of approaches, methodologies, and practice [25]. The framework is easier to calculate in parallel. The training time for tasks such as machine translation and parsing is reduced. Transformer's ability is obvious to all, and has been applied to pretraining models such as GPT, BERT, and XLM. In 2018, Brown et al. [26] proposed a unidirectional neural network language model GPT based on generative pretraining in OpenAI, which became one of the most popular pretraining models of the year. They use the fine-tuning method with two stages: the first stage uses the Transformer decoder, which is based on unlabeled corpus, for generative pretraining; the second stage is based on specific tasks for differentiated fine-tuning training, such as text classification, sentence pair relationship discrimination, text similarity, and multiple-choice tasks. Instead of adopting the traditional fully connected layers for classification in CNN, GPT directly feeds the resulting vector into the softmax layer.

Moreover, in 2018, Devlin et al. [25] proposed a pretraining model BERT based on a deep, two-way Transformer. Unlike GPT, the feature extractor used by BERT is the Transformer encoder part. Similarly, BERT is also divided into two stages, pretraining and downstream task fine-tuning. BERT changes the unidirectional language model in the GPT into a bidirectional one. Instead of using the standard left-to-right prediction of the next word as the target task, BERT proposes two new tasks. The first pretraining task is called MLM, or Masked Language Model. In the input word sequence of this model, 15% of the words are randomly masked and the task is to predict what they are. What we see is that, unlike previous models, BERT can predict these words from both directions—not just left-to-right or right-to-left. For example, Yu et al. [27] proposed a replication study of BERT pretraining that carefully measures the impact of many key hyper-parameters and training data size. Experimental results show that BERT achieved the SOTA results on GLUE, RACE, and SQuAD. Moreover, ERNIE [27] is an exploratory framework for continuous learning and understanding based on knowledge enhancement proposed by Baidu. The framework combines big data presets with multi-source knowledge. Through learning technology, it continuously absorbs knowledge of the text structure and learns in massive data texts to realize the model. ERNIE has achieved SOTA effects in more than 40 classic NLP missions, and has won more than 10 championships on international celebrities such as GLUE, VCR, XTREME, and SemEval.

UniLM is a BERT-based model, which is a simple but effective multimodal pretraining method of text. Unlike BERT, UniLM can be configured using different self-attention masks to aggregate context for different types of language models. It is made up of Transformer AI models jointly pretrained on large amounts of text and optimized for language modeling. The UniLM model uses three types of language modeling (one-way model, two-way model, and sequence-to-sequence prediction model) for pretraining [28]. Using a shared Transform network, a specific self-attention mask is used to control the context of prediction conditions, thereby achieving unified modeling. For example, in the work of [29], they proposes UniVL: a Unified Video and Language pretraining model for both multimodal understanding and generation. It comprises four components, including two single-modal encoders, a cross encoder, and a decoder with the Transformer backbone. Five objectives, including video-text joint, conditioned masked language model (CMLM), conditioned masked frame model (CMFM), video-text alignment, and language reconstruction, are designed to train each of the components. The train skills in [30–33] are applied in this paper.

In this paper, a semisupervised approach based on UniLM is proposed. The model allows unsupervised previewing and supervised tuning for language processing tasks. Experiment results show a maximum accuracy of 79.57% of the proposed model. The contributions of this paper as follows: this paper proposes a semisupervised way to deal with math word problem (MWP) tasks using unsupervised pretraining and supervised tuning methods, which are based on the Unified pretrained Language Model (UniLM). It combines the advantages of AR and AE language models to support one-way, sequence-to-sequence, and two-way prediction tasks. Experiments, carried out on MWP tasks with 20,000+ mathematical questions, show that the improved model outperforms the traditional models with a maximum accuracy of 79.57%.

The paper is structured as follows: we first introduce our methodology in Section 2, and then describe the test-bed and evaluate the proposed model according to several evaluation metrics in Section 3. After evaluating the performance of the proposed model, the summary and discussion about future work are described in Section 4.

## 3. Methodology

Researchers found that BERT could be useful for more than just Google searches [34, 35]. BERT seems to promise improvements in key areas of computational linguistics, including chat-bots, question-answering, summarization, and sentiment detection. It's defined as a “groundbreaking” technique for NLP because it's the first-ever bidirectional and completely unsupervised technique for language representation, which means a understanding of each word all at once. This represents a clear advantage in the field of context learning. It will continue revolutionizing the field of NLP because it provides an opportunity for high performance on small datasets for a large range of tasks.

**Figure 3:**
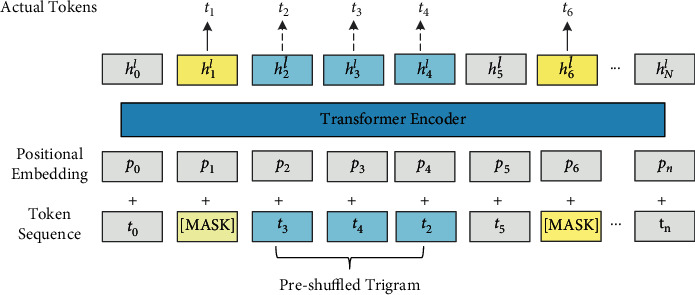
The architecture of the word structural objective.

**Figure 4:**
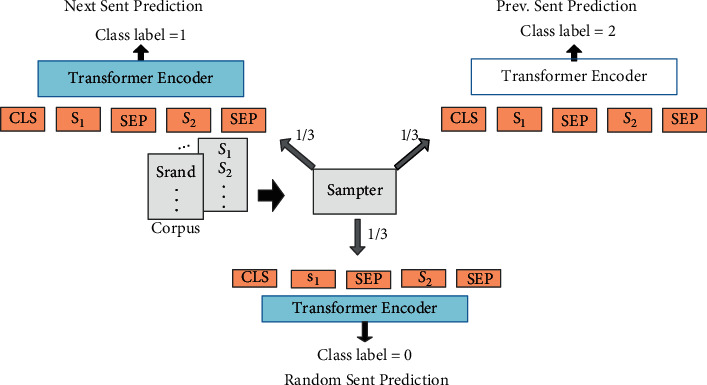
The architecture of the sentence structural objective.

The proposed model is also a multi-layer Transformer network based on UniLM, which is a BERT-based generative model. Compared to BERT, however, the proposed model can complete the three pretraining goals at the same time. Besides the mentioned pretraining methods, a new sequence-to-sequence training method is added into the model, which leads to the good performance of our model on NLU and NLG tasks. Moreover, the proposed model completes the prediction of the mask word through the context of the mask word, which is also a cloze task. For different training objectives, the context is different. The general processes of our proposed model are shown below:

(i) *Input presentation*: Each input *x* is a sequence composed of word tokens. The sequence can be either a sentence or a pair of sentences combined together. The input representation is the same as UniLM. For each input token *t*_*i*_, the *x*_*i*_ is obtained by calculating its corresponding representation through the corresponding token embedding, position embedding, and segment embedding. For the token at the beginning/end of the sequence, we add a special classification embedding (CLS)/a special end-of-sequence (SEP) of each paragraph.
(ii) *Transformer Encoder*: Then the multi-layer bidirectional Transformer encoder is used to encode the context information represented by the input. Given the input vector *X*={*x*_*i*_}_*i*=1_^*n*^ , the encoding form of an L-layer Transformer's input is as follows: *H*^*l*^=Transformer(*H*^*l*−1^) where, *l* ∈ [1, *L*], *H*^0^=*X*, *H*^*l*^=[*h*_1_^*L*^,…, *h*_*N*_^*L*^], and *H*^*l*^ is 210 the implicit vector, which is used as the contextual representation for *t*_*i*_.
  *Pretraining Objectives*: After the encoder process, we have carried out two extensions to the original UniLM pretraining goal to make full use of the rich intrasentence structure and inter-sentence structure in the language: word structure goal (mainly used for single sentence tasks) and sentence structure goal (mainly used for sentence pair tasks)). The two auxiliary targets and the original masking LM target are pretrained to find the internal language structure in a unified model. The structure is shown in Figures 3 and 4
  *Word Structural Objective*: Figure 3 shows the method of jointly training the new word target and the mask language model target. For each input sequence, first, like UniLM, we randomly mask 15% of the token, and then send the output vector to the softmax classifier to predict the original mask. Next, given a randomly scrambled token, the order of the new words is considered. The word goal is equivalent to maximizing the possibility of placing each scrambled token in the correct position. The equation can be formulated as formula fd1:
    (1) $$\begin{array}{c} \arg\underset{\theta }{\max}\sum \text{log}P\left({\text{pos}}_{1}=t_{1},{\text{pos}}_{2}=t_{2},\ldots ,{\text{pos}}_{k}=t_{k}|t_{1},t_{2},\ldots ,t_{K},\theta \right). \end{array}$$
Here, *θ* represents the trainable parameters in our model. *K* indicates the length of each scrambled subsequence. A bigger *K* will force the model to be able to reconstruct a longer sequence, while injecting more interference inputs. We take *K* = 3 to balance the model's reproducibility and robustness.
(iii) *Sentence Structural Objective*: The original UniLM model is very effective in predicting the next sentence (97%–98% accuracy rate). In our model, it is necessary to predict not only the next sentence but also the previous sentence, such that the pretrained language model perceives the order of sentences in a bidirectional manner. As shown in Figure 4, given a pair of sentences (*S*_1_, *S*_2_), where *S*_2_ may be the next sentence of *S*_1_ or not, probably speaking, there is a two-third probability that *S*_2_ is the next sentence or previous sentence of *S*_1_. Or there is a one-third probability that they are irrelevant. We use the SEP token to connect *S*_1_ and *S*_2_, and then the CLS encoded vector is input into the softmax classifier for the three-class prediction.

## 4. Experiments

In this section, we evaluate the effectiveness of the proposed model on math problems from the widely used benchmark MAWPS. MAWPS [36, 37] is an online repository of Math Word Problems and provides a unified test-bed to evaluate different algorithms. MAWPS allows for the automatic construction of datasets with particular characteristics, providing tools for tuning the lexical and template overlap of a dataset as well as for filtering ungrammatical problems from web-sourced corpora. The online nature of this repository facilitates easy community contribution. At present, the repository has amassed 3320 problems, including the full datasets used in several prominent works. Moreover, we study the effect of different parameters in our model. In the experiments, almost every possible hyper-parameter is the same for the training recipes of both models. Specifically, we carefully control the following hyper-parameters:

The same batch size: 256.

- The same number of training steps: 1*M*
- The same optimizer: Adam, learning rate 1*e* − 4, warmup 10*K*, linear decay
- The same training corpora: The dataset provides a training set containing 1674 question and answer pairs, and (251) the test set includes 865 question and answers pairs. We choose 900 questions from the 252 total training set as the development set and the remaining 1639 question and answer pairs (253) as the actual training set
- The same model architecture parameters: 24 layers, 1024 hidden size, 16 heads
- The same fine-tuning hyper-parameter search space

### 4.1. Metrics

To compare the performance of different models, Macro Precision(MP), Macro Re-call(MR), and Average F1(F1) value are adopted. The final ranking is based on average accuracy. In a corpus of *Q*_1_, *Q*_2_, *Q*_3_ …, *Q*_*N*_, the calculation of the three metrics is listed below:

Macro Precision(MP): As shown in formula (2), MP is the quotient of answers that are correctly selected and the total amount of dataset. MP will measure the accuracy of the model.
  (2) $$\begin{array}{c} MR=\frac{\left|C\right|}{\left|T\right|}. \end{array}$$
Macro Recall(MR): MR is the ratio of the number of shared words to the total number of words in the ground truth. As shown in formula (3), S is the amount of data that is predicted. It measures the completeness of the result.
  (3) $$\begin{array}{c} MR=\frac{\left|C\right|}{\left|S\right|}. \end{array}$$
F1: F1 score is a common metric for classification problems and is widely used in QA. It is appropriate when we care equally about precision and recall. The calculation is as shown in formula.
  (4) $$\begin{array}{c} F1=\frac{2}{MR^{-1}+MP^{-1}}=2\frac{MR*MP}{MR+MP}. \end{array}$$

In Table 1, it is clear that our model achieves a considerable progress in Macro Precision, Macro Recall, and F1 score. It is very hard for a model to make a huge improvement for math word problem solvers, for MWP is a mature research area.

### 4.2. Data Preparation

The dataset provides a training set containing 1674 question and answer pairs, and a test set including 865 question and answers pairs. We choose 900 questions from the total training set as the development set, and the remaining 1639 question and answer pairs as the actual training set.

### 4.3. Results

The experiment in this paper consists of two parts: Experiment 1 makes a comparison with other benchmark models. As shown in Table 1, the accuracy results of the proposed model and various baselines are listed. It is obvious that the proposed model outperforms all baselines in the experiments, and achieves a best accuracy of 79.57%. For example, in the experiment, the proposed method raised the F1 score to 0.78 compared with 0.73 and 0.76, respectively, of Graph2Tree and GTS [38]. This is because UniLM combines the advantages of both AR and AE models, which makes up for the disadvantages of LSTM, i.e., LSTM only stores information of one direction. Obviously, the proposed model performs the best in all tasks. To get a better understanding of how the constrained model is able to perform so well, we further carry experiments to test the effect of different parameters in our model.

### 4.4. Impact of the Length of the Sentence

We first study the effect of length of the sentence. The experiments are carried on the test set to investigate how the proposed model performs with increasing length of the sentence. Comparisons are built between ours and state-of-the-art models using explicit tree decoders. As shown in Table 2, we find that: First, the proposed model performs better than the other models in most of the cases, except in the case of the number of operators equals to 5. In other cases, with less than 5 operators, the model shows a good improvement compared to other models. Second, when the complexity of the sentence grows, the performance of all models decreases. This is because longer sentences lead to more complex questions, which are more difficult to predict.

### 4.5. Impact of Numerical Comparison

Since the wrong arithmetic order leads to incorrect solution expression generation, our proposed model aims to solve it. Experiments are carried to prove this by investigating how the model has improved the arithmetic order problem. We first retrieve the MWPs with incorrectly predicted expressions.

In the experiment, we check that the incorrectly predicted expressions length is equal to their corresponding ground truth expressions' length. As shown in Table 3, the proposed model gets 101 incorrect predicted sentences, while GTS has 119 and Graph2Tree has 103. We then check the amount of incorrectly predicted sentences with the initially retrieved set. The results show the same conclusion; our proposed model always generates fewer arithmetic order error sentences. This suggests that the proposed model is able to significantly improve the arithmetic order in MWP tasks.

## 5. Conclusions

This paper proposed an improved MWP model, which improves the task performance by adding UniLM for pretraining. UniLM completes unidirectional, sequence-to-sequence, and bidirectional prediction tasks. Through experiments, we show the superiority of our model against state-of-the-art models on math problem tasks.

There are three advantages of the proposed model: (1) Although there are three language model tasks in the pretraining process, we do not need to train the three models separately because the parameters of the transformer are shared. Thanks to the self-attention masking of UniLM. (2) Parameter sharing makes the learned text representation more universal because these parameters are jointly optimized with different language models. It also alleviates the problem of over-fitting on a specific language model task. (3) The proposed model is suitable for both NLU and NLG problems.

For future work, since the proposed model has difficulties dealing with long and complex sentences, we aim to consider the relationships among quantities and other attributes to better understand the context. Moreover, in future research, since advanced optimization algorithms also have been applied in many domains of NLP tasks, we may explore a comparison between advanced optimization algorithms and our model.

## Figures and Tables

**Table 1:** Comparison for math solving task.

Method	Macro precision	Macro recall	F1 score
Sedq2Seq	77.40	76.99	0.77
GTS	72.20	74.30	0.73
Graph2Tree	77.89	75.88	0.76
Our model	79.57	77.69	0.78

**Table 2:** Comparison for math solving task with different lengths of sentences.

Op	Pro	AST-Dec	GTS	Our model
1	17.4	81.5	83.1	86.2
2	51.2	74.1	79.5	84.1
3	18.4	60.1	72.1	74.2
4	6.37	43.5	52.1	53.4
5	4.30	45.6	37.6	39.4
6	0.88	56.6	46.2	56.3

**Table 3:** Comparison for arithmetic order errors.

Method	MWPs	Initially retrieve set
Seq2Seq	121	133
GTS	119	231
Graph2Tree	109	105
Our model	103	101

## Data Availability

The data used to support the findings of this study are available from corresponding author upon request.

## References

[1] Mikolov T. (2012). Statistical language models based on neural networks. *Presentation at Google, Mountain View, 2nd April 2012*.

[2] Raychev V., Vechev M., Yahav E. Code completion with statistical language models.

[3] Melis G., Dyer C., Blunsom P. (2017). On the state of the art of evaluation in neural language models. https://arxiv.org/abs/1707.05589.

[4] Kim Y., Jernite Y., Sontag D., Rush A. M. (2016). *Character-aware Neural Language Models*.

[5] Mezzoudj F., Benyettou A. (2018). An empirical study of statistical language models: n-gram language models vs. neural network language models. *International Journal of Innovative Computing and Applications*.

[6] Mnih A., Teh Y. W. (2012). A fast and simple algorithm for training neural probabilistic language models. https://arxiv.org/abs/1206.6426.

[7] Wang K., Thrasher C., Viegas E., Li X., Hsu B. j. P. An overview of Microsoft Web N-gram corpus and applications.

[8] Taher S. A., Akhter K. A., Hasan K. A. N-gram based sentiment mining for bangla text using support vector machine.

[9] Wang Y., Liu X., Shi S. Deep neural solver for math word problems.

[10] Park J., Liu X., Gales M. J., Woodland P. C. Improved Neural Network Based Language Modelling and Adaptation.

[11] Sharma M., Joshi S., Chatterjee T., Hamid R. (2022). A Comprehensive Empirical Review of Modern Voice Activity Detection Approaches for Movies and TV Shows. *Neurocomputing*.

[12] Chen X., Wang Y., Liu X., Gales M. J., Woodland P. C. Efficient GPU-Based Training of Recurrent Neural Network Language Models Using Spliced Sentence bunch.

[13] Collobert R., Weston J. A unified architecture for natural language processing: deep neural networks with multitask learning.

[14] Church K. W. (2017). Word2Vec. *Natural Language Engineering*.

[15] Pennington J., Socher R., Manning C. D. G. Glove: global vectors for word representation.

[16] Johnson R., Zhang T. (2015). Semi-supervised convolutional neural networks for text categorization via region embedding. *Advances in Neural Information Processing Systems*.

[17] Joulin A., Grave E., Bojanowski P., Douze M., Jegou H., Mikolov T. (2016). Fasttext.zip: compressing text classification models. https://arxiv.org/abs/1612.03651.

[18] Rong X. (2014). Word2vec parameter learning explained. https://arxiv.org/abs/1411.2738.

[19] Karita S., Chen N., Hayashi T. A comparative study on transformer vs rnn in speech applications.

[20] Raffel C., Shazeer N., Roberts A. (2019). Exploring the limits of transfer learning with a unified text-to-text transformer. https://arxiv.org/abs/1910.10683.

[21] Alayba A. M., Palade V., England M., Iqbal R. A combined CNN and LSTM model for Arabic sentiment analysis.

[22] Tenney I., Das D., Pavlick E. (2019). BERT rediscovers the classical NLP pipeline. https://arxiv.org/abs/1905.05950.

[23] Polignano M., Basile P., Gemmis D. M., Semeraro G., Basile V. A. Italian BERT language understanding model for NLP challenging tasks based on tweets.

[24] Kitaev N., Kaiser L., Levskaya A. R. (2020). The efficient transformer. https://arxiv.org/abs/2001.04451.

[25] Devlin J., Chang M. W., Lee K., Toutanova K. (2018). Bert: pre-training of deep bidirectional transformers for language understanding. https://arxiv.org/abs/1810.04805.

[26] Brown T. B., Mann B., Ryder N. (2020). Language models are few-shot learners. https://arxiv.org/abs/2005.14165.

[27] Yu F., Tang J., Yin W. (2020). Ernie-vil: knowledge enhanced vision-language representations through scene graph. https://arxiv.org/abs/2006.16934.

[28] Li Z., Cai J., He S., Zhao H. Seq2 seq dependency parsing.

[29] Luo H., Ji L., Shi B. (2020). Univl: a unified video and language pre-training model for multimodal understanding and generation. https://arxiv.org/abs/2002.06353.

[30] Dulebenets M. A. (2021). An Adaptive Polyploid Memetic Algorithm for scheduling trucks at a cross-docking terminal. *Information Sciences*.

[31] Li J., Wang L., Zhang J., Wang Y., Dai B. T, Zhang D. Modeling intra relation in math word problems with different functional multi-head attentions.

[32] Pasha J., Nwodu A. L., Fathollahi . (2022). Exact and metaheuristic algorithms for the vehicle routing problem with a factory-in-a-box in multi-objective settings. *Advanced Engineering Informatics*.

[33] Yu W., Wen Y., Zheng F., Xiao N. Improving math word problems with pre-trained knowledge and hierarchical reasoning.

[34] Obied Z., Solyman A., Ullah A., Fat’hAlalim A., Alsayed A. BERT Multilingual and Capsule Network for Arabic Sentiment Analysis.

[35] Sur C. R. B. N. (2020). Enhancement in language attribute prediction using global representation of natural language transfer learning technology like Google BERT. *SN Applied Sciences*.

[36] Mandal S., Naskar S. K. (2019). Solving Arithmetic Mathematical Word Problems: A Review and Recent Advancements, Advances in Intelligent Systems and Computing. *Information Technology and Applied Mathematics*.

[37] Wang L., Zhang D., Zhang J. Template-based math word problem solvers with recursive neural networks.

[38] Kitagawa H., Takenouchi T., Azuma R. (2003). Safety, pharmacokinetics, and effects on cognitive function of multiple doses of GTS-21 in healthy, male volunteers. *Neuropsychopharmacology*.

